# Subclinical dermal involvement is detectable by high frequency ultrasound even in patients with limited cutaneous systemic sclerosis

**DOI:** 10.1186/s13075-017-1270-8

**Published:** 2017-03-20

**Authors:** A. Sulli, B. Ruaro, V. Smith, S. Paolino, C. Pizzorni, G. Pesce, M. Cutolo

**Affiliations:** 1 Research Laboratory and Academic Division of Clinical Rheumatology, Department of Internal Medicine, University of Genova, Viale Benedetto XV, n° 6, AOU IRCCS San Martino, 16132 Genova, Italy; 2 Department of Rheumatology, Ghent University Hospital, Department of Internal Medicine, Ghent University, Ghent, Belgium; 3 Laboratory of Autoimmunity, Department of Internal Medicine, University of Genova, IRCCS A.O.U, San Martino, Genoa, Italy

**Keywords:** Systemic sclerosis, Dermal thickness, High-frequency ultrasound, Rodnan skin score, Nailfold videocapillaroscopy

## Abstract

**Background:**

The aim of the study was to detect by skin high-frequency ultrasound (US) possible subclinical skin involvement in patients affected by limited cutaneous systemic sclerosis (lcSSc), in those skin areas apparently not affected by the disease on the basis of a normal modified Rodnan skin score (mRSS). Differences in dermal thickness (DT) in comparison with healthy subjects were investigated.

**Methods:**

Fifty patients with lcSSc (age 62 ± 13 years (mean ± SD), disease duration 5 ± 5 years) and 50 sex-matched and age-matched healthy subjects (age 62 ± 11 years) were enrolled. DT was evaluated by both mRSS and US at the usual 17 skin areas (zygoma, fingers, dorsum of the hands, forearms, upper arms, chest, abdomen, thighs, lower legs and feet). Non-parametric tests were used for the statistical analysis.

**Results:**

Subclinical dermal involvement was detected by US even in the skin areas in patients with lcSSc, who had a normal local mRSS. In addition, statistically significantly higher mean DT was found in almost all skin areas, when compared to healthy subjects (*p* < 0.0001 for all areas). In particular, DT was significantly greater in patients with lcSSc than in healthy subjects in four out of six skin areas with a normal mRSS (score = 0) (upper arm, chest and abdomen), despite the clinical classification of lcSSc.

**Conclusions:**

This study strongly suggests that subclinical dermal involvement may be detectable by US even in skin areas with a normal mRSS in patients classified as having lcSSc. This should be taken into account during SSc subset classification in clinical studies/trials.

## Background

Systemic sclerosis (SSc) is a connective tissue disorder characterized in the early stages by microvascular damage, with progressive fibrosis and skin impairment, the latter being a marker for disease classification and activity [1–6]. Skin involvement may be recognized and studied using the modified Rodnan skin score (mRSS), the validated method used to evaluate the severity of skin thickening in SSc, and to distinguish between patients with either limited (lcSSc) or diffuse (dcSSc) cutaneous involvement [2–4, 7]. As per definition, the affected skin is confined to the extremities (hands, forearms, feet, lower legs and face) in lcSSc, whilst it is also present on upper arms, chest, abdomen and thighs in dcSSc [4].

The mRSS has some drawbacks, as it is unable to identify slight alterations in skin thickness and has high intra-observer and inter-observer variability [3, 8–10]. Conversely, several studies report the utility of high-frequency ultrasound (US) for early identification of skin involvement in patients with SSc [11–15]. US may identify the different skin layers and offers a wide range of values for measurement of dermal thickness (DT), compared with the semi-quantitative mRSS scale comprising only 4 integer values. However, mRSS and US do not measure exactly the same properties of the skin. The mRSS measures skin thickness, texture and fixation, while US accurately measures the DT, even if it is difficult to differentiate between oedema and fibrosis [11, 16, 17].

The aim of this study was to use US to detect possible subclinical skin involvement in patients with lcSSc, in those skin areas apparently not affected on the basis of a normal local mRSS, looking for differences in DT in comparison with healthy subjects.

## Methods

### Study population

Fifty patients with lcSSc (age 62 ± 13 (mean ± SD) years, mean disease duration 5 ± 5 years), classified on the basis of a normal mRSS (score = 0) at the upper arms, chest, abdomen and thighs, were enrolled during routine clinical follow up [7]. Patients with SSc met either the American College of Rheumatology (ACR)/European League Against Rheumatism (EULAR) 2013 criteria for SSc, or the LeRoy’s criteria for the classification of early SSc, and gave written informed consent to enter the study [1, 4]. Ethics approval was obtained from the local ethical board. Complete medical history was recorded, and clinical examination was carried out in all patients (the most important clinical findings are reported in Table 1).

**Table 1:** Clinical findings in patients with systemic sclerosis (SSc) and healthy control subjects (CNT)

	Age (years)	BMI (kg/m^2^)	ANA pattern (cen/spe + nuc/spe)	ENA (Scl70/RNAP/neg)	RP duration (years)	SSc duration (years)	MES (score)	US-DT total (mm)	mRSS total (score)
CNT (*n* = 50) mean ± SD	64.9 ± 15.1	22.3 ± 1.9	-	-	-	-	0	14.7 ± 0.5	0
lcSSc (*n* = 50) mean ± SD	62.5 ± 12.7	22.4 ± 1.8	33/11/6	11/1/5	12.1 ± 11.6	5.3 ± 4.9	3.2 ± 2.5	17.2 ± 1.7	4.8 ± 2.6
SSc vs CNT *p* value*	n.s.	n.s.	-	-	-	-	-	<0.0001	<0.0001
Early (*n* = 21) mean ± SD	61.3 ± 12.8	22.3 ± 2.0	14/5/2	5/0/2	6.6 ± 4.5	3.1 ± 3.5	0.7 ± 0.5	16.2 ± 1.0	3.2 ± 1.8
Active (*n* = 16) mean ± SD	58.6 ± 9.6	22.1 ± 1.5	12/2/2	3/1/0	13.0 ± 13.4	4.3 ± 3.7	4.4 ± 1.3	17.7 ± 1.2	5.0 ± 2.2
Late (*n* = 13) mean ± SD	69.2 ± 14.0	23.0 ± 2.3	7/4/2	3/0/3	20.0 ± 13.0	10.0 ± 5.0	6.0 ± 1.1	18.2 ± 2.0	7.3 ± 2.4
E vs A vs L *p* value**	n.s.	n.s.	n.s.	n.s.	0.002	0.0002	<0.0001	0.0005	<0.0001

*RP* Raynaud’s phenomenon, *MES* microangiopathy evolution score, *DT* dermal thickness (ultrasound evaluation), *mRSS* modified Rodnan skin score, *SD* standard deviation, *E* Early, *A* Active, *L* Late (patterns of microangiopathy on nailfold videocapillaroscopy), *lcSSc* limited cutaneous systemic sclerosis, *BMI* body mass index, *ANA* antinuclear antibodies, *cen* centromeric, *spe + nuc* speckled and nucleolar, *spe* speckled, *ENA* extractable nuclear antigen antibodies, *Scl70* anti-topoisomerase antibodies, *RNAP* anti-RNA polymerase III autoantibodies, *neg* ENA-negative. *Mann-Withney *U* test. **Kruskal-Wallis test

Treatments received by patients included mainly aspirin, vasodilators, immunomodulatory drugs and endothelin-1 receptor inhibitors; there were no restriction criteria related to therapy for inclusion of patients in the study, due to the cross-sectional nature of the investigation and the limited presence of possible bias in the primary endpoint.

Fifty sex-matched and age-matched healthy control subjects (CNT) (mean age ± SD 62 ± 13 years) were also evaluated after giving informed consent. US was performed in both patients with SSc and in healthy subjects, as described subsequently. Patients and healthy subjects with presence of lower extremity oedema, which could confound both mRSS and US assessment, were excluded.

### Skin high-frequency ultrasound

**Fig. 1:**
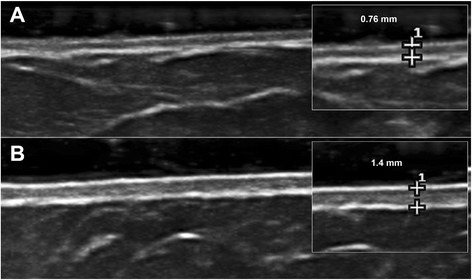
Example of measurement of dermal thickness by skin high-frequency ultrasound (18 MHz probe) in a healthy subject (**a**) and in a patient with systemic sclerosis (**b**) at the level of the abdomen

Skin US was performed in both patients with lcSSc and in healthy subjects to measure DT at the level of all 17 skin areas that are evaluated by the mRSS (at exactly the same spots), and the values were recorded in millimetres [12, 13] (see Fig. 1).

An ultrasound system equipped with an 18-MHz probe was used (MyLab 25, Esaote, Genoa, Italy). A high-frequency probe offers considerably good resolution, allowing the distinction between the epidermis, dermis and subcutaneous layers of skin, and measurement of DT [18]. In particular, DT was measured on the B-mode image by an electronic caliper included with the software, identifying the upper surface epidermis-dermis and the lower layer dermis-subcutis [12]. The same operator (BR) performed the US evaluations in all individuals, blinded to the mRSS.

### Modified Rodnan skin score

The severity of skin involvement was quantified by the mRSS in each individual. It was performed in the codified seventeen skin areas (zygoma, fingers, dorsum of the hands, forearms, upper arms, chest, abdomen, thighs, lower legs and feet) [7]. Skin thickness was assessed by palpation, and marked on a scale as 0 (normal), 1 (weak), 2 (intermediate) or 3 (severe skin thickening). In this study only patients classified as affected by lcSSc were enrolled (mRSS = 0 at the arms, chest, abdomen and thighs). The same operator (SP) assessed the mRSS in all subjects, blinded to the US assessment. Both mRSS and US were performed the same day in all patients with SSc.

### Nailfold videocapillaroscopy

Nailfold videocapillaroscopy (NVC) was performed using an optical probe, equipped with a × 200 contact lens, connected to image analysis software (Videocap, DS Medica, Milan, Italy) to classify patients with SSc into the proper pattern of microangiopathy (as early, active or late), and to calculate the microangiopathy evolution score (MES), as previously reported [19–22]. The same operator (CP) performed all NVC evaluations.

### Statistical analysis

Non-parametric tests were used for the statistical analysis. In particular, the Mann-Whitney *U* test was performed to compare unpaired groups of variables, and the Kruskal-Wallis test was used to compare continuous variables with nominal variables with more than two levels. The Spearman rank correlation test was employed to identify relationships between variables, along with linear regression tests. *P* values lower than 0.05 were considered statistically significant. The results are reported as mean with standard deviation (SD) and confidence intervals (CI).

## Results

**Fig. 2:**
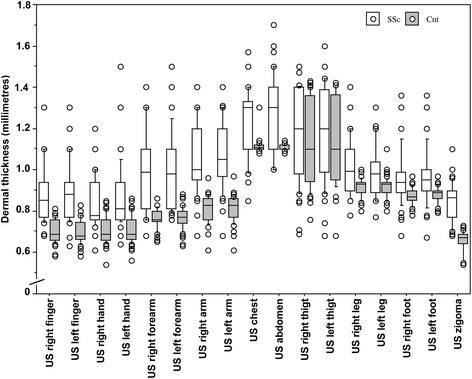
Dermal thickness evaluated by skin high-frequency ultrasound (*US*) in patients with systemic sclerosis (*SSc*) and healthy control subjects (*CNT*) (SSc vs CNT: *p* < 0.0001 for all, with the exclusion of thigh). Data are presented as box plots for different skin areas, with the 5th, 10th, 50th (median), 90th, 95th percentiles

The clinical features of patients with SSc and healthy subjects are reported in Table 1. Subclinical dermal involvement was detected by US even in areas of skin areas in patients with lcSSc who had local normal mRSS in those areas. When compared with healthy subjects, patients with lcSSc had a statistically significant higher mean DT in all skin areas (*p* < 0.0001 for all) except the thighs, where DT was greater in patients with lcSSc than in healthy subjects but the difference was not statistically significant (*p* = 0.16 and *p* = 0.14, respectively for the right and left thigh) (see Table 2 and Fig. 2 for further statistical data).

**Table 2:** Dermal thickness in healthy subjects and patients classified as affected by limited cutaneous systemic sclerosis (lcSSc) on the basis of a normal Rodnan skin score at the upper arms, chest, abdomen and thighs

Dermal thickness	Healthy subjects					Patients with lcSSc	Patients with lcSSc with DT >2SD	Patients with lcSSc with DT >3SD
	Mean ± SD (mm)	Mean + 2SD (mm)	Mean + 3SD (mm)	95% CI lower, upper	99.73% CI lower, upper	Mean ± SD (mm)	Number (%) (out of 50)	Number (%) (out of 50)
Right finger	0.70 ± 0.05	0.80	0.85	0.69, 0.72	0.68, 0.72	0.88 ± 0.14	32 (64)	25 (50)
Left finger	0.70 ± 0.06	0.82	0.88	0.69, 0.72	0.68, 0.72	0.88 ± 0.14	32 (64)	28 (56)
Right hand	0.71 ± 0.06	0.83	0.89	0.69, 0.72	0.68, 0.73	0.84 ± 0.12	23 (46)	13 (26)
Left hand	0.71 ± 0.06	0.83	0.89	0.69, 0.73	0.69, 0.74	0.86 ± 0.16	24 (48)	18 (36)
Right forearm	0.77 ± 0.05	0.87	0.92	0.75, 0.78	0.75, 0.79	0.98 ± 0.19	32 (64)	28 (56)
Left forearm	0.77 ± 0.05	0.87	0.92	0.75, 0.78	0.75, 0.79	0.99 ± 0.19	34 (68)	30 (60)
Right upper arm	0.82 ± 0.07	0.96	1.03	0.80, 0.83	0.79, 0.84	1.06 ± 0.16	37 (74)	23 (46)
Left upper arm	0.82 ± 0.06	0.94	1.00	0.80, 0.83	0.79, 0.84	1.07 ± 0.16	39 (78)	25 (50)
Chest	1.11 ± 0.03	1.17	1.20	1.11, 1.12	1.10, 1.13	1.23 ± 0.17	35 (70)	24 (48)
Abdomen	1.11 ± 0.02	1.15	1.17	1.11, 1.12	1.11, 1.12	1.28 ± 0.18	37 (74)	37 (74)
Right thigh	1.13 ± 0.21	1.55	1.76	1.08, 1.18	1.05, 1.21	1.18 ± 0.23	0	0
Left thigh	1.14 ± 0.20	1.54	1.74	1.08, 1.19	1.05, 1.22	1.18 ± 0.23	1 (2)	0
Right lower leg	0.92 ± 0.04	1.00	1.04	0.91, 0.93	0.90, 0.93	1.03 ± 0.13	25 (50)	16 (32)
Left lower leg	0.92 ± 0.05	1.02	1.07	0.90, 0.93	0.90, 0.93	1.03 ± 0.10	21 (42)	16 (32)
Right foot	0.87 ± 0.04	0.95	0.99	0.86, 0.88	0.86, 0.89	0.96 ± 0.13	24 (48)	17 (34)
Left foot	0.88 ± 0.04	0.96	1.00	0.87, 0.89	0.86, 0.89	0.97 ± 0.11	25 (50)	15 (30)
Zygoma	0.66 ± 0.05	0.76	0.81	0.65, 0.67	0.64, 0.68	0.85 ± 0.10	38 (76)	32 (64)

Mean values, standard deviations (SD) and both 95% and 99.73% confidence intervals (CI) are reported. Of interest, patients with lcSSc had dermal thickness (DT) values greater than the normal range in healthy subjects (see mean +2SD and +3SD, and upper 95% and 99.73% CI reporting, respectively, the 5% and 0.27% chance that healthy subjects might have a dermal thickness above the range), also in those areas where the mRSS was zero

Of interest, DT was also significantly higher in four out of six skin areas where the mRSS was normal (score = 0) (upper arms, chest and abdomen). Moreover, at the level of the upper arms, chest and abdomen the mean DT in patients with lcSSc was higher than mean DT plus three standard deviations in the healthy subjects, and thus was above the normal range (99.73% probability) (see Table 2 for CI).

In particular, almost 75% of patients with SSc had DT beyond the normal range in the aforementioned skin areas (instead of the expected 5% of patients allowing for possible variation from the normal range), despite their classification as having lcSSc; furthermore, 46–74% of patients had DT above the 99.73% CI upper limit in the aforementioned skin areas) (see also Table 2).

As was predictable, the sum of the DT values for the 17 areas of skin assessed by either US or the mRSS was significantly higher in patients with SSc than in the control group (Table 1). There was statistically significant positive correlation between total US-DT and mRSS-DT values (*r* = 0.37, *p* = 0.04).

There was no statistically significant correlation between DT and the duration of either SSc (*p* = 0.7) or Raynaud’s phenomenon (RP) (*p* = 0.6). Neither was there any statistically significant correlation between DT and organ involvement (gastrointestinal tract, lung, heart, kidney or occurrence of digital ulcers) in our cohort of patients with lcSSc.

Patients with lcSSc who were positive for anti-centromere antibodies had lower DT (17.07 ± 1.65 mm) than patients with anti-Scl-70 (17.78 ± 1.89 mm) or anti-RNA polymerase III (19.30 ± 0.0 mm), but the difference was not statistically significant (*p* = 0.40) (however the population was small and unbalanced in terms of the autoantibody profile). Dermal thickness, as evaluated by both US and the mRSS, was significantly higher in those patients with lcSSc who had the “late” pattern of microangiopathy on NVC and an elevated microangiography evolution score (MES) (see Table 1). The intra-operator reproducibility was 92% (95%CI 0.87–0.96) for mRSS and 96% (95% CI 0.94–0.97) for US.

## Discussion

The present investigation demonstrates, for the first time, that skin high frequency US is able to identify subclinical dermal involvement even in the skin areas where the mRSS is normal, in patients classified as affected by lcSSc. Skin involvement in SSc is critical for the initial diagnosis and it also has prognostic relevance [2, 3].

Cutaneous manifestations are clinically recognized and studied by the mRSS, the validated method to assess the severity of skin involvement in SSc [5, 7, 9]. The extension of skin involvement, evaluated by mRSS, is the parameter for classification of the disease into different subsets, characterized by either limited or diffuse skin involvement [2, 4]. This classification is largely used in clinical trials/studies.

Patients with lcSSc may have increased DT at the extremities, forearms, lower legs and face, but not at the upper arms, chest, abdomen and thighs, which characterizes the patients affected by dcSSc, as assessed by mRSS [2, 3, 7]. Therefore, the present US study of the skin brings important information to be considered when the patients are classified as being affected by lcSSc.

The subclinical skin involvement in patients with lcSSc is further supported in the present study by showing that mean DT values were beyond the normal DT value of the sex-matched and age-matched healthy control subjects (calculated as mean DT value plus three standard deviations, 99.73% CI upper limit): about 50% of individual patients had DT values above the 99.73% CI upper limit, and about 75% above the 95% CI upper limit (standard normal range) at the arms, chest and abdomen.

These observations seem to have a genetic and pathophysiological background, as recent studies carried out by gene microarray analysis suggested that in patients with SSc the clinically unaffected skin shares peculiar gene signatures and pathological aspects, similar to the overt clinically affected skin [23, 24]. More recently, clustering analysis revealed two prominent transcriptomes in skin biopsies from patients with SSc: the keratin and fibro-inflammatory signatures [25]. Interestingly, in both patients with dcSSc and patients with lcSSc, hyalinised collagen and myofibroblasts were identified even in skin that was not clinically involved, without significant differences between lcSSc and controls [26, 27].

Further possible applications of integrating skin US analysis with the mRSS in patients with SSc could originate from the recent observation that the baseline mRSS was the strongest predictor of skin improvement, independent of disease duration [28]. These findings also seem to link to the report that patients with either lcSSc or dcSSc may display similar organ/laboratory involvement in clinical studies [29–32]. Altogether these reports seem to suggest that US may identify skin involvement earlier than the mRSS in apparently unaffected skin areas in patients with lcSSc. This is also supported by other studies, which demonstrated that US is able to identify the oedematous phase preceding palpable skin involvement in the early stage of SSc [11, 12, 18].

One limitation to the present study might be the small cohort of enrolled patients, due to having recruitment at a single centre, and the larger standard deviation in DT observed in both patients and healthy subjects at the thighs might justify the absence of statistically significant differences between the two groups at this level. Furthermore, the US evaluation was made by only one operator, and so inter-rater reliability was not assessed.

Another limitation might be the 18-MHz probe employed to assess DT, due to its good but sub-optimal performance in analysing the skin; modern higher frequency probes (20–24 MHz) may allow easier identification of the dermal boundaries, reducing measurement error. Once again, the greater variation in DT observed at the thighs might be related to a slightly blurred image obtained with our 18-MHz probe at this level.

A further limitation may be linked to the fact that the patients with SSc were analysed without considering therapeutic management; however, the aim of the present study was to assess possible subclinical skin involvement in individual patients with lcSSc and ongoing treatments should not influence the results. Finally, DT may vary at different ages and according to premenopausal or postmenopausal status [33]; however, this bias was avoided by enrolling sex-matched and age-matched subjects.

By considering the capability of US to detect skin involvement in the early and subclinical stages of SSc, skin US might be proposed as a further important tool for the clinical assessment of the disease. Of note, skin US may be considered a notable and acceptable technique for clinical research into the pathogenesis of the disease and treatment effects, as it represents a non-invasive and safe approach [34].

As this was a cross-sectional study, data on future worsening of DT (evaluated by both the mRSS and US) or progression from lcSSc to dcSSc are not provided. Further studies should investigate this matter. In terms of the feasibility of skin US, it is more time-consuming than mRSS assessment as it takes about 20–25 minutes including skin image capture and manipulation to measure DT. However, the examination is well-accepted by patients, and it do not imply further expense if the device is the same already employed to assess musculoskeletal apparatus during routine clinical practice.

## Conclusions

In conclusion, this study strongly suggests that subclinical dermal involvement may be detectable by skin high-frequency ultrasound even in patients classified as having lcSSc on the basis of the mRSS clinical evaluation. This should be taken into consideration during subset classification in clinical studies/trials.

## Abbreviations

CI: Confidence intervals
CNT: Controls, healthy subjects
dcSSc: Diffuse cutaneous systemic sclerosis
DT: Dermal thickness
IQR: Interquartile range
lcSSc: Limited cutaneous systemic sclerosis
MES: Microangiopathy evolution score
mRSS: Modified Rodnan skin score
NVC: Nailfold videocapillaroscopy
SD: Standard deviation
SSc: Systemic sclerosis
US: High-frequency ultrasound
